# Cost Burden of Rigid Internal Fixation in Craniomaxillofacial Trauma Care in Low‐ and Middle‐Income Countries

**DOI:** 10.1002/oto2.92

**Published:** 2023-11-05

**Authors:** David A. Shaye, Obinna Nwosu, Isaie Ncogoza, Victor Nyabyenda, Gratien Tuyishimire, Wayne Manana, Abdurrazaq Olanrewaju Taiwo

**Affiliations:** ^1^ Division of Facial Plastic and Reconstructive Surgery, Department of Otolaryngology–Head and Neck Surgery, Massachusetts Eye and Ear Harvard Medical School Boston Massachusetts USA; ^2^ Department of Surgery University Teaching Hospital of Kigali Kigali Rwanda; ^3^ Department of Oral and Maxillofacial Surgery, Faculty of Health Sciences University of Zimbabwe Harare Zimbabwe; ^4^ Departments of Oral and Maxillofacial Surgery/Dental and Maxillofacial Surgery, Faculty of Dental Sciences Usmanu Danfodiyo University/Usmanu Danfodiyo University Teaching Hospital Sokoto Nigeria

**Keywords:** cost, craniomaxillofacial trauma, low‐income countries, middle‐income countries, open reduction internal fixation

## Abstract

Fractures of the craniomaxillofacial (CMF) skeleton cause significant morbidity and mortality in low‐ and middle‐income countries (LMICs). Despite this, quality CMF trauma care is lacking for the majority of the world's population. There is a paucity of literature describing the costs of standard‐of‐care open reduction internal fixation (ORIF) for CMF fractures in LMICs. We consider the cost of a six‐hole plate with six screws (SHPS), standard materials used in ORIF for CMF fractures, as a percentage of gross domestic product (GDP) per capita to ascertain the cost burden to patients. Hospital pricing catalog data at 14 LMIC institutions were queried. On average, the SHPS cost represented 10.2% of the GDP per capita in sampled LMICs. We highlight manufacturing costs, import taxes, and lack of subsidized health care as factors contributing to the significant cost burden of ORIF in these areas. Future work should characterize additional financial and socioeconomic barriers to optimal CMF care.

Global morbidity and mortality from trauma exceed that of HIV/AIDS, malaria, and tuberculosis combined, yet trauma and its management garner little comparative attention.^1^ Craniomaxillofacial (CMF) fractures represent a significant component of overall trauma in low‐ and middle‐income countries (LMICs).^2^ Open reduction internal fixation (ORIF) is the gold standard for CMF fractures, but patients in LMICs must purchase their own plating supplies. As a result, less than 20% of these patients undergo ORIF for CMF fractures.^3^

Prior work has broadly described the state of CMF care in LMICs; however, there is a paucity of literature describing the costs of ORIF in LMICs.^4^ A focused review on ORIF costs can help to identify specific opportunities for decreasing financial barriers. We aim to characterize ORIF material cost burden by considering the cost of a single six‐hole plate and six screws (SHPS), standard ORIF materials, as a proportion of gross domestic product (GDP) per capita.

## Methods

A group of 14 established CMF surgeons, currently practicing in LMICs, were queried as to the costs of the select ORIF materials at their respective hospitals. Cost to patients was extracted from hospital catalog pricing data. Costs were similarly obtained from local academic centers in the Boston area for comparative analysis. LMICs are defined as countries with a gross national income per capita, as determined by the World Bank in 2019, of less than $12,375.^5^ GDP per capita, as reported by the World Bank in 2019, was used as a metric of average individual income.^5^ All costs are reported in 2019 US dollars. The cost burden is defined as the cost of the select ORIF materials expressed as a proportion of GDP per capita. Data analysis was performed in Excel (Microsoft Corp). This study was exempt from the Mass General Brigham Institutional Review Board.

## Results

The mean SHPS cost among the 14 sampled LMICs was $238.38. The cost was lowest in Rwanda ($4.79) and highest in Gabon ($1000). The mean cost burden (SHPS cost/GDP per capita) among sampled LMICs was 10.2%. The cost burden was lowest in Rwanda (0.6%) and highest in Cameroon (23.2%). The mean SHPS cost at 3 local Boston academic centers was $692.21; this represents 1.1% of GDP per capita (Table 1).

**Table 1:** Costs of a Single Six‐Hole Plate With Six Screws, GDP Per Capita, and Calculated Cost Burden for Sampled Low‐ and Middle‐Income Countries and Local Boston Academic Centers

Country	Cost (USD)	GDP per capita (USD)	Cost burden,^a^ %
Rwanda	$4.79	$820.03	0.6
Egypt	$27.00	$3019.21	0.9
Eswatini^b^	$100.00	$3894.68	2.6
South Africa	$307.48	$6001.40	5.1
Nigeria	$120.00	$2229.86	5.4
Pakistan	$75.00	$1284.70	5.8
Namibia	$105.71	$4957.46	2.1
Zimbabwe	$125.00	$1463.99	8.5
Gabon	$1000.00	$7767.01	12.9
Ghana	$312.00	$2202.12	14.2
Zambia	$235.00	$1305.06	18.0
Kenya	$387.00	$1816.55	21.3
Ethiopia	$188.30	$855.76	22.0
Cameroon	$350.00	$1507.45	23.2
Average (n = 14)	$238.38	$2794.66	10.2
Local Academic Center			
BCH	$687.19	$65,297.52	1.1
BMC	$801.80	$65,297.52	1.2
MEE/MGH	$587.63	$65,297.52	0.9
Average (n = 3)	$692.21	$65,297.52	1.1

Abbreviations: BCH, Boston Children's Hospital; BMC, Boston Medical Center; GDP, gross domestic product; MEE, mass eye and ear; MGH, Massachusetts General Hospital; USD, US dollars.

^a^ Cost burden is defined as the cost divided by the GDP per capita.

^b^ Eswatini was formerly known as Swaziland.

## Discussion

The majority of global trauma occurs in LMICs, causing significant morbidity and mortality.^1^ Elucidating barriers to CMF trauma care is imperative to improving access and patient outcomes.^6^ We show that the mean cost burden of SHPS was 10.2% (range 0.6%‐23.2%) in the sampled LMICs. This cost burden is multifactorial with manufacturing costs, import taxes, and lack of subsidized health care playing critical roles.

Manufacturing titanium, which is used for ORIF materials because of its strength and biocompatibility, is a resource‐intensive process. Consequently, titanium is more expensive than other more readily available metals in LMICs.^4^ In high‐income countries, the use of bioresorptive materials has shown promise in decreasing overall trauma costs by reducing reoperative rates, without compromising patient outcomes.^7^ Such innovations offer opportunities and could help to cut costs in LMICs.^8^ However, similar to titanium, these materials are not commonly available in LMICs.

Manufacturing costs alone cannot explain SHPS costs in LMICs. The highest SHPS cost reported in our study was in Gabon. This may be due to high import taxes which limit the supply of medical devices to Gabon and other LMICs.^9^ The cost of importing medical devices in Gabon contributes to the nation's striking total health care costs which represent 21.8% of its GDP per capita.^10^ SIGN International is a nonprofit organization that coordinates donations of ORIF materials for hip surgery to LMICs.^11^ Development of similar donation platforms for CMF materials may help offset costs.

Interestingly, with the exception of Gabon, SHPS costs were lower in all sampled LMICs than in Boston centers. This may be due to intrinsic market limits in LMICs which cannot tolerate costs as steep as US markets can. While SHPS costs in LMICs were lower, the cost burden was significantly higher when compared to Boston. This burden is exacerbated by the comparative rarity of medical insurance in LMICs. Where most patients in the United States are shielded from a significant proportion of the material cost by insurance, only a minority of LMICs have national medical insurance programs that subsidize costs.^12^ Indeed, health care system refinancing is needed in LMICs.

This study has notable limitations. ORIF material costs represent an important but limited portion of the barriers to CMF care. Other financial costs (ie, surgeon/anesthesia fees, perioperative care, transportation costs), shortage of CMF surgeons, lack of reliable, efficient referral patterns, and socioeconomic and environmental factors were not considered. Additional research should characterize such factors and their impact on access to CMF care in LMICs. While a more comprehensive review of access to CMF care would be valuable, a targeted review of the cost burden of ORIF materials may help to guide practical solutions for decreasing costs. Separately, data regarding median household income was not universally available from all countries. Thus, GDP per capita, a measure of total domestic economic output per person, was used as a proxy for median income. GDP per capita is a broad measure and lacks the specificity requisite for considering cost burden. In resource‐limited settings where national surveys are not easily conducted, this may be the most robust measure of median income available.

## Conclusion

Our study demonstrates the significant cost burden of standard CMF care in LMICs. Future work should detail other barriers and focus on the development of innovative solutions for improving access.

## Author Contributions

**David A. Shaye**, study design, data acquisition, manuscript drafting/editing, analysis; **Obinna Nwosu**, manuscript drafting/editing, analysis; **Isaie Ncogoza**, design; **Victor Nyabyenda**, design; **Gratien Tuyishimire**, study design; **Wayne Manana**, study design; **Abdurrazaq Olanrewaju Taiwo**, study design, data acquisition, manuscript drafting/editing.

## Disclosures

### Competing interests

None.

### Funding source

None.

## References

[1] World Health Organization . Injuries and Violence: The Facts. World Health Organization; 2014.

[2] Ozgursoy OB , Muderris T , Yorulmaz I , Kucuk B . Demographic, epidemiologic, and surgical characteristics of maxillofacial fracture repair in a developing country. Ear Nose Throat J. 2009;88(4):20‐24. 19358115

[3] Taiwo A , Godwin N , Ibikunle A , Soyele O . Facial fracture management in northwest Nigeria. J Surg Tech Case Rep. 2013;5(2):65‐71. 10.4103/2006-8808.128723 24741422 PMC3977327

[4] Shah I , Gadkaree SK , Tollefson TT , Shaye DA . Update on the management of craniomaxillofacial trauma in low‐resource settings. Curr Opin Otolaryngol Head Neck Surg. 2019;27(4):274‐279. 10.1097/MOO.0000000000000545 31274568

[5] World Bank. World Bank national accounts data. 2019. Accessed April 15, 2021. https://data.worldbank.org/indicator/NY.GDP.PCAP.CD

[6] Whitaker J , Nepogodiev D , Leather A , Davies J . Assessing barriers to quality trauma care in low and middle‐income countries: a Delphi study. Injury. 2020;51(2):278‐285. 10.1016/j.injury.2019.12.035 31883865

[7] Gilardino MS , Chen E , Bartlett SP . Choice of internal rigid fixation materials in the treatment of facial fractures. Craniomaxillofac Trauma Reconstr. 2009;2(1):49‐60. 10.1055/s-0029-1202591 22110797 PMC3052646

[8] DePasse JW , Caldwell A , Santorino D , et al. Affordable medical technologies: bringing Value‐Based Design into global health. BMJ Innov. 2016;2(2):4‐7. 10.1136/bmjinnov-2015-000069

[9] Hakobyan S , Cherif R . Trade in Medical Goods: Challenges and a Way Forward for Sub‐Saharan Africa. International Monetary Fund; 2021.

[10] Obiang Obounou BW . Gabon health care system: a 20 year analysis. Int J Community Med Public Health. 2017;4(7):2208‐2211. 10.18203/2394-6040.ijcmph20172807

[11] Gillard J , Smith T . A model for sustainable orthopedic care in low and middle income countries. ASME International Mechanical Engineering Congress and Exposition, Proceedings (IMECE). ASME; 2021:5. 10.1115/IMECE2020-23332

[12] Hooley B , Afriyie DO , Fink G , Tediosi F . Health insurance coverage in low‐income and middle‐income countries: progress made to date and related changes in private and public health expenditure. BMJ Glob Health. 2022;7(5):e008722. 10.1136/bmjgh-2022-008722 PMC9092126 35537761

